# Potential benefits of combining transfluthrin-treated sisal products and long-lasting insecticidal nets for controlling indoor-biting malaria vectors

**DOI:** 10.1186/s13071-018-2811-y

**Published:** 2018-04-10

**Authors:** John P. Masalu, Fredros O. Okumu, Arnold S. Mmbando, Maggy T. Sikulu-Lord, Sheila B. Ogoma

**Affiliations:** 1 Environmental Health and Ecological Sciences Department, Ifakara Health Institute, Off Mlabani Passage, Ifakara, Morogoro, United Republic of Tanzania; 2 School of Public Health, University of the Witwatersrand, Johannesburg, South Africa; 3 Institute of Biodiversity, Animal Health and Comparative Medicine, University of Glasgow, Glasgow, UK; 4 Queensland Alliance of Agriculture and Food Innovation, The University of Queensland, Brisbane, Australia; 5 US National Research Council, National Academies of Sciences, Engineering, and Medicine, Washington, D.C., USA

**Keywords:** Residual malaria transmission, Spatial repellents, LLINs, Early-evening biting, Transfluthrin

## Abstract

**Background:**

Transfluthrin vapour prevents mosquito bites by disrupting their host-seeking behaviors. We measured the additional benefits of combining transfluthrin-treated sisal decorations and long-lasting insecticidal nets (LLINs) with an aim of extending protection against early evening, indoor-biting malaria vectors when LLINs are ineffective.

**Methods:**

We investigated the indoor protective efficacy of locally made sisal decorative baskets (0.28 m^2^) treated with 2.5 ml and 5.0 ml transfluthrin, in terms of mosquito density, exposure to bites and 24 h mortality. Experiments were conducted in experimental huts, located in Lupiro village, Ulanga District, south-eastern Tanzania. Human landing catches (HLC) were used to measure exposure to bites between 19:00–23:00 h. Each morning, at 06:00 h, mosquitoes were collected inside huts and in exit traps and monitored for 24 h mortality.

**Results:**

Sisal decorative baskets (0.28 m^2^) treated with 2.5 ml and 5.0 ml transfluthrin deterred three-quarters of *Anopheles arabiensis* mosquitoes from entering huts (relative rate, RR = 0.26, 95% confidence interval, CI: 0.20–0.34, *P* < 0.001 and RR= 0.29, 95% CI: 0.22–0.37, *P* < 0.001, respectively). Both treatments induced a 10-fold increase in 24 h mortality of *An. arabiensis* mosquitoes (odds ratio, OR = 12.26, 95% CI: 7.70–19.51, *P* < 0.001 and OR = 18.42, 95% CI: 11.36–29.90, *P* < 0.001, respectively).

**Conclusions:**

Sisal decorative items treated with spatial repellents provide additional household and personal protection against indoor biting malaria and nuisance mosquitoes in the early evening, when conventional indoor vector control tools, such as LLINs, are not in use. We recommend future studies to investigate the epidemiological relevance of combining LLINs and transfluthrin decorated baskets in terms of their effect on reduction in malaria prevalence.

## Background

Long-lasting insecticidal nets (LLINs), indoor residual spraying (IRS), improved diagnosis and treatment have brought about substantial decline in malaria transmission, particularly in sub-Saharan Africa [1–3]. Despite these achievements, residual malaria transmission that occurs even with high coverage of LLINs and/or IRS continues to threaten efforts towards malaria elimination. Additionally, insecticide resistance in Africa is another challenge in consolidating, and sustaining the gains accrued by vector control tools [4–7].

Effectiveness of LLINs depends on factors that influence human-vector contact, such as time and place of malaria-transmitting mosquito bites [8], user’s sleeping hours, proper use, installation and maintenance of nets, as well as user’s compliance [9]. When LLINs are not available, the risk of exposure to infectious bites increases during meal times, at social events and or when students are doing homework. In addition, in rural Africa most people live in houses that are not sufficiently proofed to prevent mosquito entry [10].

Topical repellents [11, 12] and protective clothing [13] represent some of the options used as personal protection against mosquito bites when LLINs are not in use. Although these tools confer some protection, they have some limitations: (i) they divert mosquitoes to non-users [14]; (ii) they require reapplication often hourly; and (iii) they often fail due to non-compliance by users [15]. Additionally, topical repellents are unlikely to be practical for daily use, and may not be affordable for continuous use in low and middle-income populations [16]. Due to high temperatures in some regions, and costs required for re-application, the use of protective clothing may not be feasible in most tropical countries. Development of new, efficacious, low-cost, context specific, practical and scalable vector control tools, that target indoor biting mosquitoes when LLINs are not in use, would complement the protective efficacy of LLINs and IRS.

Spatial repellents are vapour-phase insecticides that incapacitate mosquitoes and prevent them from locating hosts and obtaining blood meals [17]. Examples of spatial repellent delivery formats include pyrethroid-treated mosquito coils, vaporizer mats, aerosols, and paper strips as well as traditional practices such as burning and smoldering plants [18].

Previous studies have shown that transfluthrin prevents mosquitoes from feeding [19], and induces mosquito mortality [20]. Here, we quantified the potential benefits of combining spatial repellent with LLINs, as a complementary strategy against indoor biting mosquitoes in the early evening, when LLINs are not in use.

## Methods

### Study area

The study was conducted in Lupiro village (8.385°S, 36.670°E), Ulanga District, south-eastern Tanzania [21]. Annual rainfall is 1200–1600 mm with temperature ranging between 20–32.6 °C [21]. The main malaria vectors in this area are *An. arabiensis* and *Anopheles funestus* (*sensu lato*) [22]. The main vector control intervention in the area is LLINs, with a first universal mass LLINs distribution campaign conducted between 2010 and 2011 [23, 24]. A more recent LLINs mass campaign was conducted between 2015 and 2016 [25]. Preceding studies indicated that both *An. arabiensis* and *An*. *funestus* were pyrethroid (i.e. permethrin: 77% and 65%, respectively) resistant [26] and findings from a more recent study indicated that *An*. *funestus* (*s.l.*) was also resistant to pyrethroids (i.e. permethrin: 10.5%) [27].

### Preparation of transfluthrin-treated sisal fabrics

Circular pieces of sisal 0.28 m^2^ were treated with either 2.5 ml or 5 ml of 97% transfluthrin (Shenzhen Sunrising Industry Company, Limited, Shenzhen, China) following the method previously described [28–30]. Control pieces were soaked in a mixture of water and detergent only as previously described [28–30]. All pieces were enclosed in colorfully beaded iron welded baskets as previously described [30].

### Rationale for delivering transfluthrin using sisal decorative baskets

Sisal fabrics are versatile products from the sisal plant, available in most of the tropical countries like Tanzania. These fabrics can be made into various household products, such as mats, baskets, curtains, wall picture frame, etc. The uniqueness of the sisal fabrics are: (i) they have relatively high absorbance of liquid such as water; and (ii) they allow slow release of transfluthrin in air, this way transfluthrin-treated sisal fabrics may remain effective for a duration of more than six months or a year [28, 31]. Nevertheless, as the sisal products fits for different households decorative items, using these items indoor, when are treated with transfluthrin, may serve two purposes: decorate house and act as an indoor vector control tool.

### Study design

**Fig. 1:**
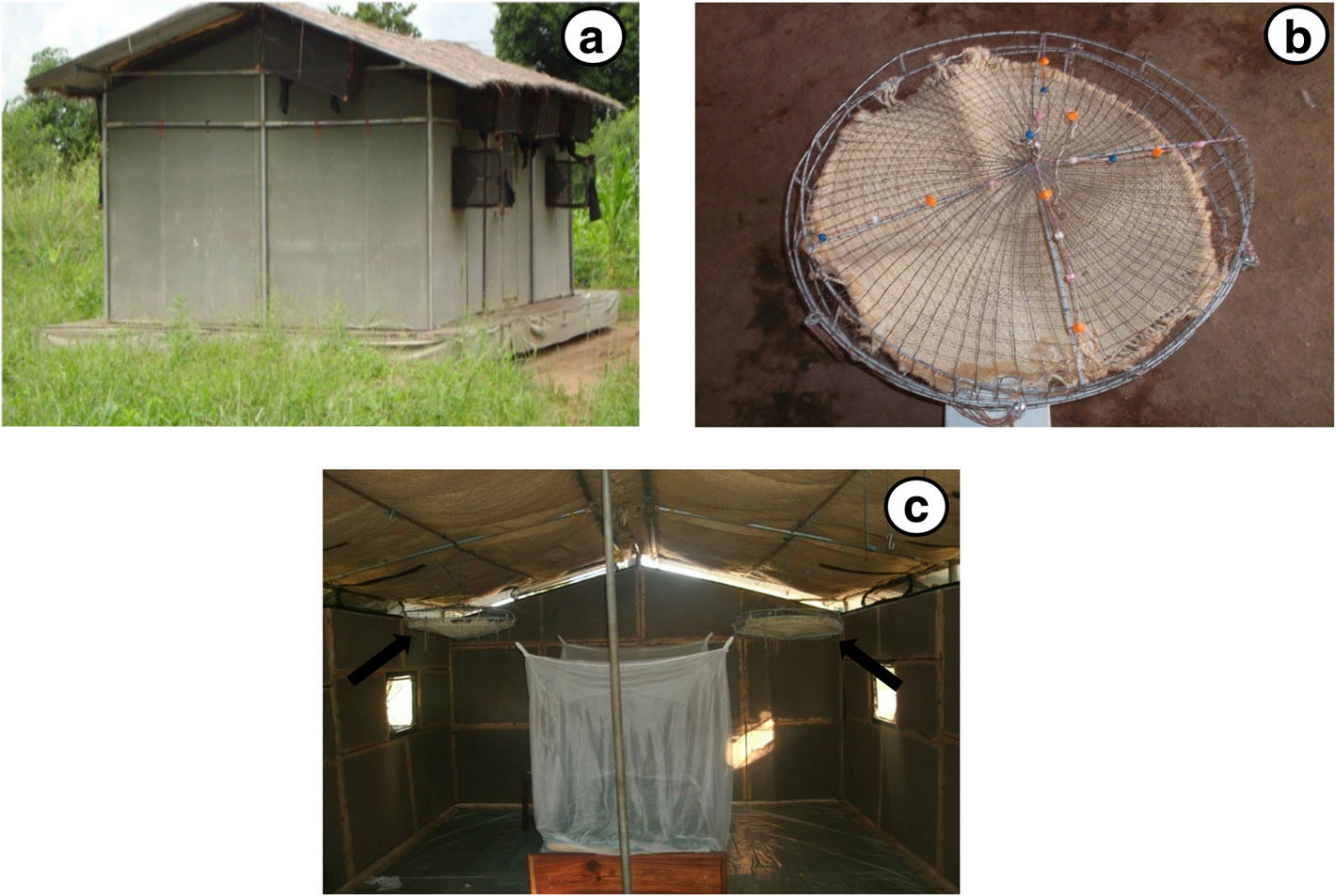
Outside view of Ifakara experimental hut design and sisal baskets decorative prototype as previously described [30]. **a** An outside view of the Ifakara experimental hut. **b** A sisal decorative basket [30]. **c** Inside view of Ifakara experimental hut with suspended sisal decorative baskets about 1.8 m from the floor and 0.52 m from the wall. The arrow indicates the position of the suspended sisal decorative basket

Experiments were conducted from 6th January 2015 to 7th February 2015. The effect of combining transfluthrin-treated sisal baskets and permethrin-treated LLINs on the proportion of indoor mosquito density, the proportion of early evening indoor mosquito bites and survival of mosquitoes in experimental huts (Fig. 1a) was investigated. The treatments included: (i) control arm with permethrin-treated LLIN and four untreated sisal baskets; (ii) four transfluthrin-treated (2.5 ml) sisal baskets and one permethrin-treated LLIN; and (iii) four transfluthrin-treated sisal baskets (5 ml) and one permethrin-treated LLIN. Initially, treatments were randomly allocated to 3 experimental huts, using a lottery method and later treatment and control arms were rotated between 3 huts after 9 consecutive experimental nights using a 3 × 3 Latin square design. A sisal basket (Fig. 1b) [30], was suspended in each of the four corners of the huts (Fig. 1c). They were placed 1.84 m off the ground and 0.52 m from the wall. In each hut, a male volunteer conducted human landing catches from 19:00 to 23:00 h. This coincided with the time when most people within this community are likely to be awake but not protected by LLINs. Moreover, mosquitoes were also collected from exit traps, fitted on eaves and windows of the huts as well as on the floor at 06:00 h. All mosquitoes were kept in a field insectary situated approximately 50 m from the nearest experimental hut. The temperature in the experimental huts was 26.94 °C during the day and 25.65 °C at night and relative humidity was 81.0% during the day and 86.5% at night. Mosquitoes were provided 10% glucose solution for 24 h after which mortality was recorded. After 24 h, mosquitoes were sorted and recorded as dead, live, blood-fed or unfed. Morphological identification keys [32] were used to identify mosquitoes to their genus and species. Standard polymerase chain reaction (PCR) [33, 34] was used to differentiate a subsample of sibling species of *An. gambiae* (*s.l.*) and *An. funestus* (*s.l.*) mosquitoes that were randomly selected each day. The primary outcomes measured included: (i) mosquito deterrence, which is reduction in the density of indoor mosquitoes; (ii) indoor human mosquito biting rate, which is the proportion of mosquitoes that landed and attempted to bite volunteers that were conducting HLC; and (iii) insecticide-induced 24 h mortality.

### Data analysis

Deterrence was determined statistically using log-normal Poisson generalized linear mixed effects models (GLMMs) in R statistical software version 3.1.3, with *lme4* package [35]. The response variable was the total number of mosquitoes collected from experimental huts including those collected indoors by those conducting HLC. Experimental huts and day of experiment were treated as random independent variables, while treatment was coded as a fixed variable. An over-dispersion random variable accounting for the random fluctuating nature of mosquito count data on different experimental days was included. *An. arabiensis*, *An. funestus* (*s.l.*) and *Culex* species mosquitoes were analyzed in separate models. The same analysis was used to measure reduction in the proportion of biting mosquitoes in the early evening. The total number of mosquitoes collected by HLC in experimental huts between 19:00 and 23:00 h was fitted as the dependent variable. The hut and the day of experiment were treated as random variables, while treatment arm was coded as a fixed variable. Insecticide induced mortality was determined by fitting a GLMMs with a binomial distribution and a logit-link function. The proportion of dead and live mosquitoes was coded as dependent binomial variable, treatment arms as fixed variable whereas day of experiment and experimental huts were treated as random variables.

## Results

The total number of mosquitoes collected was 7125. These included 4157 *Culex* spp.; 1672 *An. arabiensis*; 1165 *An. funestus* (*s.l.*); 121 *Mansonia* spp.; 4 *Coquilettidia* spp.; 3 *An. coustani*; and 3 *Aedes* spp. Of 91 *An. gambiae* (*s.l.*) samples amplified by PCR, all of the 86% (*n* = 78) successful amplifications achieved were *An. arabiensis*. Sixty-eight *An. funestus* (*s.l*.) samples were analyzed by PCR, and 71% (49/68) were successful amplifications. Of the successful amplifications, 96% (47/49) were *An. funestus* (*sensu stricto*), 2% (1/49) were *An. leesoni*, and the remaining 2% (1/49) were *An. rivulorum.*

### Deterrence

**Fig. 2:**
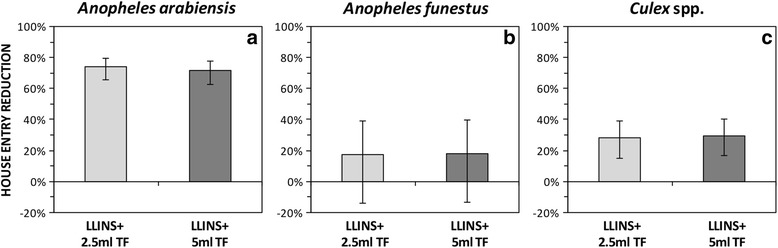
Mean indoor entry rate (mosquitoes caught per hut per night) of *An. arabiensis* (**a**), *An. funestus* (**b**) and *Culex* spp*.* (**c**) between the huts that had transfluthrin-treated sisal baskets and LLINs to those with untreated counterparts and LLINs. The error bars represent the 95% confidence intervals, CI. *Abbreviations*: LLINs, long-lasting insecticidal nets; TF, transfluthrin

Relative to LLINs with untreated sisal baskets, sisal decorative baskets treated with 2.5 ml and 5.0 ml transfluthrin, in combination with permethrin LLINs, reduced almost three quarters of indoor *An. arabiensis* mosquitoes (2.5 ml: RR = 0.26, 95% confidence interval, CI: 0.2–0.34, *P* < 0.001) and (5.0 ml: RR = 0.29, 95% CI: 0.22–0.37, *P* < 0.001) (Table 1, Fig. 2). Adding either 2.5 ml or 5 ml transfluthrin-treated baskets to LLIN huts, did not reduce indoor densities of *An. funestus* (*s.l.*) mosquitoes (2.5 ml: RR = 0.83, 95% CI: 0.60–1.14, *P* < 0.230; and 5.0 ml: RR = 0.82, 95% CI: 0.6–1.13; *P* < 0.240). Huts with transfluthrin-treated sisal baskets and LLINs had nearly one third less *Culex* sp. mosquitoes compared to those with LLINs and untreated baskets (RR = 0.72, 95% CI: 0.61–0.85, *P* < 0.001) for 2.5 ml transfluthrin and (RR = 0.70, 95% CI: 0.6–0.83, *P* < 0.001) for 5 ml transfluthrin. As shown in Fig. 2 and Table 4, there were no differences in effect between the 2.5 ml and 5 ml treatments in reducing indoor mosquito entry.

**Table 1:** Comparison of the mean mosquito entry per hut per night between huts with trasnfluthrin-treated sisal baskets in combination with LLINs to those with untreated sisal baskets and LLINs

Treatment	*n*	Mean number (adjusted)	95% CI	RR	95% CI	*P*-value
*Anopheles arabiensis*						
Untreated bd + LLINs	981	30.34	17.42–52.84	na^a^	na	na
2.5 ml bd + LLINs	292	7.86	4.45–13.86	0.26	0.19–0.34	< 0.001
5.0 ml bd + LLINs	399	8.65	4.89–15.28	0.29	0.22–0.37	< 0.001
*Anopheles funestus*						
Untreated bd + LLINs	448	13.37	9.88–18.09	na	na	na
2.5 ml TF bd + LLINs	356	11.07	8.15–15.03	0.83	0.60–1.14	0.243
5.0 ml TF bd + LLINs	361	11.01	8.11–14.94	0.82	0.59–1.13	0.230
*Culex* spp.						
Untreated bd + LLINs	1951	39.55	24.31–64.33	na	na	na
2.5 ml TF bd + LLINs	1103	28.35	17.41–46.16	0.72	0.61–0.85	< 0.001
5.0 ml TF bd + LLINs	1103	27.80	17.07–45.27	0.70	0.59–0.83	< 0.001

*Abbreviations*: *n* total number of mosquitoes collected, *CI* confidence interval, *RR* relative rate, *bd* basket decoration, *LLINs* long-lasting insecticidal net and *TF* transfluthrin

^a^na = 1 was used as a reference

### Indoor human mosquito biting rate

**Fig. 3:**
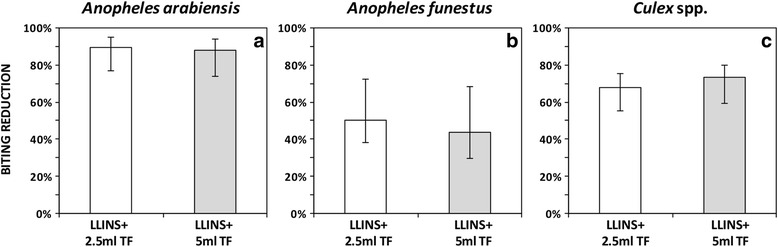
Mean biting rate (biting per person per night) against indoor bites of *An. arabiensis* (**a**), *An. funestus* (**b**) and *Culex* spp. (**c**) between the huts that had transfluthrin-treated sisal baskets and LLINs to those with untreated counterparts and LLINs. The error bars represent the 95% confidence intervals, CI. *Abbreviations*: LLINs, long-lasting insecticidal nets; TF, transfluthrin

Figure 3 and Table 2 show that both 2.5 ml and 5.0 ml transfluthrin-treated baskets, combined with LLINs, reduced the proportion of *An. arabiensis* mosquito bites by more than three quarters (2.5 ml: RR = 0.10, 95% CI: 0.05–0.23, *P* < 0.001; and 5.0 ml: RR = 0.12, 95% CI: 0.06–0.26, *P* < 0.001) compared to LLINs with untreated baskets. In addition, the two interventions reduced *An. funestus* (*s.l.*) mosquitoes bites by nearly half (2.5 ml: RR = 0.48, 95% CI: 0.27–0.87, *P* < 0.016 and 5 ml: RR = 0.56, 95% CI: 0.31–0.98, *P* < 0.043). The addition of transfluthrin-treated baskets reduced exposure to *Culex* spp. mosquitoes by approximately two thirds (2.5 ml and LLINs: RR = 0.33, 95% CI: 0.25–0.42, *P* < 0.001; and 5 ml and LLINs: RR = 0.27, 95% CI: 0.21–0.35, *P* < 0.001). Furthermore, as shown in Fig. 3 and Table 5, there were no differences in effect between the 2.5 ml and 5 ml treatments in reducing indoor mosquito biting rate.

**Table 2:** Comparison of the mean mosquito collection per person per night between huts with trasnfluthrin-treated sisal baskets in combination with LLINs to those with untreated sisal baskets and LLINs

Treatment	*n*	Mean number (adjusted)	95% CI	RR	95% CI	*P*-value
*Anopheles arabiensis*						
Untreated bd + LLINs	281	5.54	2.40–12.78	na^a^	na	na
2.5 ml TF bd + LLINs	41	0.57	0.22–1.49	0.10	0.05–0.23	< 0.001
5.0 ml TF bd + LLINs	68	0.67	0.26–1.75	0.12	0.06–0.26	< 0.001
*Anopheles funestus*						
Untreated bd + LLINs	68	1.60	0.93–2.76	na	na	na
2.5 ml TF bd + LLINs	34	0.78	0.42–1.44	0.48	0.27–0.87	0.016
5.0 ml TF bd + LLINs	37	0.89	0.49–1.61	0.56	0.31–0.98	0.043
*Culex* spp.						
Untreated bd + LLINs	523	11.89	6.99–20.21	na	na	na
2.5 ml TF bd + LLINs	149	3.89	2.24–6.75	0.33	0.25–0.42	< 0.001
5.0 ml TF bd + LLINs	111	3.20	1.84–5.58	0.27	0.21–0.35	< 0.001

*Abbreviations*: *n* total number of mosquitoes collected, *CI* confidence interval, *RR* relative rate, *bd* basket decoration, *LLINs* long-lasting insecticidal net and *TF* transfluthrin

^a^na = 1 was used as a reference

### Insecticide-induced 24 h mortality

**Fig. 4:**
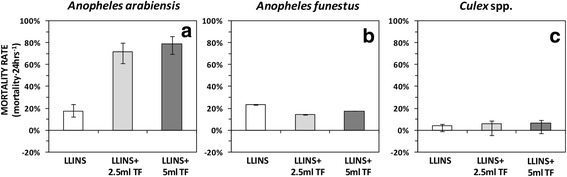
Mortality rate (mortality per 24 h post-exposure) of *An. arabiensis* (**a**), *An. funestus* (**b**) and *Culex* spp. (**c**) between the huts that had transfluthrin-treated sisal baskets and LLINs to those with untreated counterparts and LLINs. The error bars represent the 95% confidence intervals, CI. *Abbreviations*: LLINs, long-lasting insecticidal net, TF, transfluthrin

Adding transfluthrin treated baskets in experimental huts with LLINs, induced a 10-fold increase in 24 h mortality of *An. arabiensis* (OR = 12.26, 95% CI: 7.70–19.51, *P* < 0.001 for 2.5 ml and OR = 18.43, 95% CI: 11.36–29.90, *P* < 0.001 for 5 ml). Compared to the control arm, adding transfluthrin-treated sisal baskets in experimental hut with LLINs, did not have impact on inducing mortality of *An. funestus* (*s.l.*) mosquitoes 24 h post-exposure (OR = 0.54, 95% CI: 0.53–0.54, *P* < 0.001 and OR = 0.69, 95% CI: 0.69–0.70, *P* < 0.001, respectively). Neither 2.5 ml (1.57, 95% CI: 0.95–2.57, *P* < 0.076 ) nor 5.0 ml (OR = 1.67, 95% CI: 0.98–2.86, *P* < 0.061) transfluthrin-treated baskets combined with LLINs increased mortality of *Culex* spp. mosquitoes (Table 3, Fig. 4). Additionally, as shown in Fig. 4 and Table 6, there were no differences in effect between the 2.5 ml and 5 ml treatments in inducing mosquito mortality rate.

**Table 3:** Mosquito mortality after 24 h post-collection between huts with trasnfluthrin-treated sisal baskets in combination with LLINs to those with untreated sisal baskets and LLINs

Treatment	*n*	Mean proportional (adjusted)	95% CI	OR	95% CI	*P*-value
*Anopheles arabiensis*						
Untreated bd + LLINs	168	0.17	0.12–0.23	na^a^	na	na
2.5 ml TF bd + LLINs	202	0.72	0.61–0.80	12.26	7.70–19.51	< 0.001
5.0 ml TF bd + LLINs	296	0.79	0.69–0.86	18.43	11.36–29.90	< 0.001
*Anopheles funestus*						
Untreated bd + LLINs	122	0.23	0.23–0.23	na	na	na
2.5 ml TF bd + LLINs	77	0.14	0.14–0.14	0.54	0.53–0.54	< 0.001
5.0 ml TF bd + LLINs	74	0.17	0.17–0.18	0.69	0.69–0.69	< 0.001
*Culex* spp.						
Untreated bd + LLINs	87	0.03	0.02–0.05	na	na	na
2.5 ml TF bd + LLINs	70	0.05	0.04–0.08	1.57	0.95–2.57	0.076
5.0 ml TF bd + LLINs	70	0.06	0.04–0.09	1.67	0.98–2.86	0.061

*Abbreviations*: *n* total number of dead mosquitoes collected, *OR* odd ratios, *CI* confidence interval, *bd* basket decoration, *LLINs* long-lasting insecticidal net and *TF* transfluthrin

^a^na = 1 was used as a reference

## Discussion

Here, we investigated the complementary effects of transfluthrin treated baskets combined with LLINs in terms of mosquito deterrence, biting rate and 24 h mortality. We show that transfluthrin treated baskets provided comprehensive protection against *An. arabiensis* than *An. funestus* or *Culex* spp.

Long-lasting insecticidal nets confer protection *via* a range of modes of action, including excito-repellency, induced mortality of mosquitoes as well as providing physical barrier [36]. However, the emergence of insecticide resistance is undermining the benefits of LLINs and efforts towards malaria elimination [4–7]. Changing biting behavior and residual malaria transmission [37–39] have also significantly reduced the outputs of LLINs and IRS, which calls for complementary strategies.

A combination of transfluthrin-treated sisal baskets and LLINs reduced the overall numbers of indoor density of *An. arabiensis* mosquitoes by three quarters, compared to LLINs with untreated sisal baskets (Table 1, Fig. 2). However, this reduction was not observed for *An. funestus* and for *Culex* spp. Preceding studies, for example Hill et al. [40], demonstrated that a combination of transfluthrin-treated mosquito coil and LLINs resulted in massive reduction of indoor mosquito densities. Similarly, Ogoma et al. [20] demonstrated that combination of transfluthrin-treated coils and LLINs resulted in reduction of indoor mosquito densities. These studies suggest the combination of transfluthrin-based spatial repellents and LLINs may reduce the number of mosquitoes entering dwellings, thereby reducing the risk of malaria transmission.

Our findings support a most recently developed mathematical model, which suggested that combining a highly-toxic insecticide and an efficacious repellent could combat insecticide resistance while protecting people from mosquito bites [41].

Secondly, transfluthrin-treated sisal baskets reduced exposure to early evening bites of *An. arabiensis* mosquitoes, where LLINs alone may not have been effective (Table 2, Fig. 3). A similar effect, albeit lower, was observed with *An. funestus* and *Culex* spp. Didzie et al. [12] demonstrated a dramatic reduction in indoor mosquitoes bites when LLINs were used in combination with topical repellent (NO MAS, a water-based lotion with its principle active ingredient para-methane-diol and lemongrass). Similarly, Syafruddin et al. [42] demonstrated that a combination of LLINs and topical repellent (picaridin, KBR3023, SC Johnson, Racine, WI, USA) reduced indoor mosquito biting rates. The risk of malaria transmission is highest before bed time, considering the fact that LLINs will not be in use at that time. Spatial repellents that provide protection to multiple people in a wide area would be a complementary strategy to LLNs [43].

Mathematical models applied in previous studies postulate that a combination of repellent and LLINs attenuate community-wise benefit by diverting the vectors away from lethal, insecticide treated surfaces [44]. Surprisingly, a 10-fold increase in mortality of *An. arabiensis* was observed when transluthrin was used in combination with LLINs (Table 3, Fig. 4). Previously, Ogoma et al. [20] also demonstrated an increase in mortality of *An. arabiensis* and *An. gambiae* (*s.s*.) in the presence of transfluthrin coils. However, we did not observe any added benefits of combining transfluthrin decorated baskets with LLINs in terms of inducing mortality of *An. funestus* and *Culex* spp. The low mortality observed for *An. funestus* may be partly explained by pyrethroid resistance exhibited by these mosquitoes as demonstrated previously [26], and confirmed recently [27]. The findings from this study indicate that the efficacy of both 2.5 ml and 5.0 ml 97% transfluthrin treatments was similar (Tables 4, 5 and 6, Figs. 2, 3 and 4). Therefore, a lower dose is recommended for use in future studies.

**Table 4:** Comparison of the relative mosquito entry per hut per night between huts with 2.5 ml and 5.0 ml trasnfluthrin-treated sisal baskets in combination with LLINs

Treatment	RR	95% CI	*P*-value
*Anopheles arabiensis*			
2.5 ml TF bd + LLINs	na^a^	na	na
5 ml TF bd + LLINs	1.05	0.81–1.36	0.713
*Anopheles funestus*			
2.5 ml TF bd + LLINs	na	na	na
5 ml TF bd + LLINs	0.99	0.73–1.34	0.943
*Culex* spp.			
2.5 ml TF bd + LLINs	na	na	na
5 ml TF bd + LLINs	0.93	0.77–1.11	0.432

*Abbreviations*: *RR* relative rate, *bd* basket decoration, *CI* confidence interval, *LLINs* long-lasting insecticidal net and *TF* transfluthrin

^a^na = 1 was used as a reference

**Table 5:** Comparison of the relative mosquito collection per person per night between huts with 2.5 ml and 5.0 ml trasnfluthrin-treated sisal baskets in combination with LLINs

Treatment	RR	95% CI	*P*-value
*Anopheles arabiensis*			
2.5 ml TF bd + LLINs	na^a^	na	na
5 ml TF bd + LLINs	1.30	0.43–3.92	0.637
*Anopheles funestus*			
2. 5 ml TF bd + LLINs	na	na	na
5.0 ml TF bd + LLINs	1.22	0.74–2.01	0.446
*Culex* spp.			
2.5 ml TF bd + LLINs	na	na	na
5.0 ml TF bd + LLINs	0.80	0.57–1.14	0.215

*Abbreviations*: *RR* relative rate, *CI* confidence interval, *bd* basket decoration, *LLINs* long-lasting insecticidal net and *TF* transfluthrin

^a^na = 1 was used as a reference

**Table 6:** Comparison of mosquito mortality after 24 h post-exposure between huts with 2.5 ml and 5.0 ml trasnfluthrin-treated sisal baskets in combination with LLINs

Treatment	OR	95% CI	*P*-value
*Anopheles arabiensis*			
2.5 ml TF bd + LLINs	na^a^	na	na
5 ml TF bd + LLINs	1.42	0.85–2.37	0.183
*Anopheles funestus*			
2.5 ml TF bd + LLINs	na	na	na
5 ml TF bd + LLINs	1.23	0.71–2.12	0.455
*Culex* spp.			
2.5 ml TF bd + LLINs	na	na	na
5 ml TF bd + LLINs	1.01	0.61–1.65	0.979

*Abbreviations*: *OR* odd ratios, *CI* confidence interval, *bd* basket decoration, *LLINs* long-lasting insecticidal net and *TF* transfluthrin.

^a^na = 1 was used as a reference

Combining transfluthrin-treated household decorations and permethrin-treated LLINs was beneficial, and potentially enhanced protection by LLINs, against indoor biting malaria vectors by reducing indoor mosquito density and biting rate and increasing 24 h mortality. Transfluthrin is a pyrethroid, and its efficacy was less pronounced on suspected pyrethroid resistant *An*. *funestus* (*s.l.*). This calls for frequent insecticide susceptibility tests to monitor emergence of resistance.

## Conclusions

Here, we have demonstrated that transfluthrin-treated emanators combined with LLINs reduce indoor mosquito entry and protect people against indoor mosquito bites when LLINs are not in effect. The emanators increase mortality of major malaria vectors in the area. Future studies should focus on measuring epidemiological endpoints of these combined interventions.

## Abbreviations

GLMMs: Generalized linear mixed effects models
HLC: Human landing catch
IRS: Indoor residual spraying
IHI: Ifakara Health Institute
IRB: Institutional review board
LLINs: Long-lasting insecticidal nets
mRDT: Malaria rapid diagnostic test
NIMR: National Institute for Medical Research
OR: Odds ratio
PCR: Polymerize chain reaction
RR: Relative rate
TF: Transfluthrin
